# Flavonoids: New Frontier for Immuno-Regulation and Breast Cancer Control

**DOI:** 10.3390/antiox8040103

**Published:** 2019-04-16

**Authors:** Meenakshi Sudhakaran, Sagar Sardesai, Andrea I. Doseff

**Affiliations:** 1 Department Physiology, Michigan State University, East Lansing, MI 48824, USA; 2 Physiology Graduate Program, Michigan State University, East Lansing, MI 48824, USA; sudhaka7@msu.edu; 3 Division of Hematology, Department of Internal Medicine, The Ohio State University, Columbus, OH 43210, USA; 4 Department Pharmacology and Toxicology, Michigan State University, East Lansing, MI 48824, USA

**Keywords:** cancer therapeutics, cancer prevention, metastasis, chemoprevention, flavonoids, immune-regulation, apigenin, obesity, foods for health, inflammation, macrophages

## Abstract

Breast cancer (BC) remains the second most common cause of cancer-related deaths in women in the US, despite advances in detection and treatment. In addition, breast cancer survivors often struggle with long-term treatment related comorbidities. Identifying novel therapies that are effective while minimizing toxicity is critical in curtailing this disease. Flavonoids, a subclass of plant polyphenols, are emerging as promising treatment options for the prevention and treatment of breast cancer. Recent evidence suggests that in addition to anti-oxidant properties, flavonoids can directly interact with proteins, making them ideal small molecules for the modulation of enzymes, transcription factors and cell surface receptors. Of particular interest is the ability of flavonoids to modulate the tumor associated macrophage function. However, clinical applications of flavonoids in cancer trials are limited. Epidemiological and smaller clinical studies have been largely hypothesis generating. Future research should aim at addressing known challenges with a broader use of preclinical models and investigating enhanced dose-delivery systems that can overcome limited bioavailability of dietary flavonoids. In this review, we discuss the structure-functional impact of flavonoids and their action on breast tumor cells and the tumor microenvironment, with an emphasis on their clinical role in the prevention and treatment of breast cancer.

## 1. Introduction

Breast cancer (BC) is the most commonly diagnosed cancer in women worldwide and the second most common cause of cancer-related mortality in women in the US [1,2]. Despite the advancement on early detection methods and intensive interventions, questions pertaining to the mechanisms underlying the aggressiveness of certain forms of cancer such as the triple negative breast cancer (TNBC) remain unanswered. Identifying additional treatments and preventive approaches continues to be critical in curtailing BC. Epidemiological studies suggest that the consumption of healthy diets, rich in fruits and vegetables, and body weight are positively associated with a reduced incidence of BC [3,4]. Flavonoids, plant polyphenols broadly present in fruits and vegetables, are emerging as promising arsenals for the prevention and treatment of cancer and other chronic inflammatory diseases [5,6,7,8,9]. Flavonoids show anti-carcinogenic, anti-metastatic and immuno-modulatory activities in cellular and preclinical animal models making them potential candidates in cancer prevention and treatment. Despite the well-established role of flavonoids as antioxidants, recent evidences show that flavonoids directly interact with proteins, making them ideal small molecules for the modulation of enzymes, transcription factors and receptors [10]. These novel properties of flavonoids propose new platforms to modulate tumor signaling, overcome chemo-resistance and re-educate the tumor microenvironment (TME). Unraveling these novel mechanisms of action of flavonoids offers great clinical opportunities. In this review, we discuss the structure-functional impact of flavonoids and their action on tumor cells and TME, with an emphasis on their clinical role in the prevention and treatment of breast cancer.

## 2. Breast Cancer

Breast cancer is a leading cause of cancer-related mortality among women worldwide. Extensively complex and heterogeneous, BC features characteristic histological patterns and diverse biological phenotypes and clinical behaviors. Advancements in high throughput techniques have shed light into providing a better understanding of this heterogeneity, molecular features and in developing new predictive and prognostic factors to support therapy [11,12]. Hierarchical gene clustering analysis and gene-expression profiling of primary breast tumors led to its molecular classification comprising of four subtypes; luminal, human epidermal growth factor receptor (HER2) enriched, normal breast-like and basal like (Table 1) [13,14,15]. While luminal (A and B) BC is characterized by the presence of hormone receptors estrogen and progesterone positive (ER+ and/or PR+) biomarkers with good prognosis, HER2 enriched are hormone receptor negative and HER2 positive BC accompanied by a relatively worse prognosis. Although normal-like and luminal A cancer types share the same biomarker status, normal-like express normal breast profiling pattern and low proliferation marker Ki67 with a moderately worse prognosis. Basal-like, which also includes TNBC, has expression patterns either lacking or having a low expression of receptors (ER−, PR−, HER2−) and a high expression of basal markers like keratins with phenotype being more aggressive in nature.

The non-metastatic disease is primarily treated with curative intent surgery with or without adjuvant radiation. The use of systemic therapy (adjuvant or neoadjuvant) is guided by tumor characteristics, patient factors and preferences. For instance, BC patients with hormone receptor positive or HER2-amplified tumors respond favorably to targeted therapies. Endocrine therapy complimentary to surgery has prolonged the disease-free survival and overall survival rate in patients with early as well as relapsed ER+ BC [16,17,18]. Despite being approved by the US-FDA (US Food and Drug Administration) as HER2 directed therapies and ameliorating the overall survival in combination with chemotherapy, many HER2+ BC patients have shown to acquire the inherent drug resistance. Advanced cancer and TNBC, which are associated with significantly worse prognosis, are more often encountered in younger women and approved chemotherapy regimens are lacking [19,20]. Currently, advanced BC is incurable and poses a significant treatment challenge with a median overall survival (OS) of five years or less especially in TNBC with no available targeted therapies [21,22,23].

The Omic platforms have increased our understanding of tumor biology, which has led to novel treatments targeting drug resistance, oncogenic pathways and TME [24,25]. Several ongoing randomized controlled trials are investigating new drug applications targeting actionable genomic mutations in advanced BC and of particular interest is the development of immune-oncology compounds in certain BC subtypes [26,27,28,29,30,31]. However, most novel treatments are not bereft of side effects. A growing number of BC survivors suffer from long-term treatment related comorbidities such as neuropathy, obesity, cognitive deficits and cardiovascular disease [32,33,34,35,36,37]. Additionally, rising health care costs and time for therapeutic drug development are significant limitations to improving patient care in oncology [38,39]. Identifying cost-effective treatments that can rapidly move from bench to bedside with minimal additional toxicity is critical.

## 3. Flavonoids Classification and Distribution

**Figure 1:**
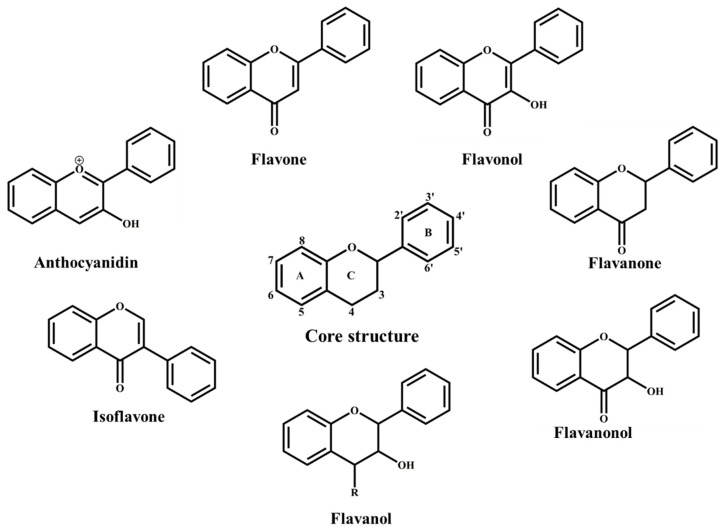
Flavonoid basic structure and classification.

Flavonoids are secondary metabolites broadly distributed in plants. The basic structure of flavonoids comprises two benzene rings (A and B) linked through a heterocyclic pyrone ring (C) [40,41]. Based on the chemical arrangements of hydroxy groups, annularity of ring C, degree of oxidation and the connection position of ring B, flavonoids are categorized into different sub-groups that include: flavones, flavonols, flavanones, flavanonols, flavanols, isoflavones and anthocyanidins (Figure 1) [41,42,43].

Flavones represent one of the largest groups and possess a functional C2=3 double bond in the basic structure. Flavonoids are often found to be hydroxylated, glycosylated (bound to sugars) or methoxylated in different positions. This diversity in the degree of substitution, polymerization, conjugation and the substitution of functional groups accounts for the wide spectrum of biological and pharmacological activities and also determines their availability in vivo.

The bioavailability of flavonoids varies among different subclasses but has shown to be quite poor in several of them, thereby being a hindrance in reaching effective concentrations in vivo. Structural features play a crucial role in determining the absorption and bioavailability of flavonoids. For example, absorption of glycosides is generally lower than the aglycone counterparts, with the rate of deglycosylation being determined by the number and type of sugars [44,45]. We showed that the presence of sugars reduces the cellular absorption of flavones, limiting the immuno-modulatory activity of glycosides as compared to aglycones and thereby affecting their bioavailability in vivo [46]. The type of glycosylation affects the hydrolysis of flavonoids in the intestine. The hydrolysis of C-glycosyl flavonoids for example, is less effective as compared to O-glycosides. In contrast to the hydroxylated forms, O-methylated flavonoids possess better bioavailability owing to its delayed absorption and increased permeability across membranes [46,47,48,49]. There is a paucity of data on the relationship between the structure activity and biological fate of the flavonoids. Notwithstanding their broad distribution, the bioavailability of flavonoids is poor and demands the establishment of beneficial dietary recommendations. The emerging importance of the flavonoids in human diet and health calls for the need to further evaluate the relationship between the structure and function and their impact in cancer biology.

## 4. Flavonoids: Molecular Mechanisms of Action

### 4.1. Antioxidant Effects of Flavonoids in Breast Cancer

The health benefits of the flavonoids have been historically ascribed to their chelating and antioxidant properties led by their innate chemical structure [50]. The presence of multiple hydroxyl groups along with a highly conjugated delocalized electron system enhance the free radical scavenging nature of the flavonoids and its interference with the redox activity of the cell [51]. In general, flavonoids have been shown to block the production of reactive oxygen species (ROS) in macrophages, thereby mediating their immune modulating activity [52,53,54,55].

In vitro studies on hormone sensitive ER+ human BC T47D and MCF-7 cell lines have shown that flavonoids found in wine, like quercetin and resveratrol, inhibit proliferation by antagonizing hydrogen peroxide (H_2_O_2_) [56]. Flavones possessing multiple hydroxyl groups, such as apigenin and luteolin, induced apoptosis and cell cycle arrest in MCF-7 and the TNBC cell lines through the inhibition of PI3K/Akt and increased FOXO3a (forkhead box protein) activation, which is associated with ROS reduction [57]. Butein, a tetrahydroxy chalcone, suppressed the growth of HCC70 (TNBC), BT-474 (HER2+) and T47D (ER+) human BC cell lines by reducing ROS and inducing apoptosis, as evidenced by the increased caspase-3 and caspase-9 activities [58]. Interestingly, flavonoids can also induce ROS accumulation, depending on the cell type and culture conditions, suggesting additional mechanisms responsible for triggering cell death. The flavanone naringenin induces ROS dependent apoptosis in human MDA-MB-468 TNBC cells [59]. Myricetin can suppress the growth of MDA-MB-231 and MDA-MB-468 cell lines by activating pro-apoptotic agents via a pro-oxidant effect, which is mediated by H_2_O_2_ generation [60,61,62]. The flavone 5,7-dihydroxy, 8-nitrochrysin, exerted cytotoxicity on HER2 overexpressing MDA-MB-453 human BC cells by the generation of ROS and Akt dephosphorylation [63,64]. Silibinin, a mixture of flavanolignans, induced apoptosis in MDA-MB-231 cells through ROS-dependent Notch-1/ERK/Akt pathway and nuclear translocation of the apoptosis-inducing factor (AIF) [65]. Flavonoids can also induce apoptosis in a ROS-independent pathway. Common shortcoming in the field is that sometimes the observed changes in activities represent secondary effects that might not be necessary and/or sufficient for the biological effects exerted by the flavonoids. Illustrating this point, our group showed that the flavone apigenin induces a quick ROS production followed by caspase-3-dependent apoptosis in leukemia cells. However, we found that ROS inhibitors failed to inhibit apoptosis, clearly indicating that ROS production was dispensable for the apigenin-induced cell death [66]. Therefore, the beneficial effects of flavonoids in cellular models of BC can be due to antioxidant-dependent and independent pathways. Hence, caution on the interpretation of the results and studies devoted to a deeper dissection of the mechanisms of action are much needed in the field of flavonoids for health.

In BC xenograft mouse models, flavonoids have shown to significantly reduce cell growth through the production and scavenging of ROS. The myricetin derived flavonoid oncamex, induced increased superoxide production and cytotoxicity in MDA-MB-231 xenografts [67]. Treatment of BT-474 xenografts with butein reduced tumor growth through the reduction of ROS and induction of apoptosis [58]. Flavonoids extracted from *Radix Glycyrrhiza* attenuated tumor mass of MDA-MB-231 xenografts by the inhibition of iNOS (inducible Nitric Oxide Synthase) and inactivation of the JAK2/STAT3 signaling pathway [68]. Apigenin and luteolin inhibited intravasation mechanisms in the MDA-MB-231 breast cancer spheroids thereby hindering its growth and migration through the lymph endothelial barrier [69]. This study reveals insights into the potential effects of flavonoids to inhibit metastasis. Future studies using 3D cultures of BC cell lines and with patient derived xenografts (PDX) will be crucial for evaluating the clinical potential of flavonoids alone or in combination with chemotherapeutic drugs in BC treatment.

BC recurrence, metastasis, and the limited success of current therapies are in part ascribed to the complexity of the TME. Macrophages, key immune cells abundant in the TME, regulate tumor growth, invasion, metastasis and harbor immune-suppressive conditions [70,71]. Increased numbers of tumor associated macrophages (TAMs) in the TME and circulatory myeloid progenitors, including inflammatory monocytes (i-Mo) and myeloid derived suppressor cells (MDSCs), are responsible for defective immune-surveillance [72,73,74]. The presence of these cells has been correlated with increased metastasis and poor clinical prognosis [75]. There is also compelling evidence that TAMs limit the efficacy of chemotherapy [76,77,78,79]. Thus, the ability of flavonoids to target the macrophage function has attracted great interest as a potential approach to increase the efficacy of current standard of care and reduce the immunosuppressive conditions of the TME in BC.

We showed that the flavone apigenin exerts immunoregulatory activity in macrophages and its myeloid progenitors. Apigenin reduces the phosphorylation of p65 subunit of nuclear factor NFκB, responsible for its transcriptional activity [52]. NFκB is a key transcription factor in cancer controlling both the proliferation of cancer cells and the immune response [80]. NFκB regulates the expression of tumor necrosis factor alpha (TNFα) and interleukin-1β (IL-1β) in macrophages, whereas in tumor cells, increases the expression of anti-apoptotic molecules [81], contributing to increased resistance of cancer cell to current chemotherapies. A study involving multiple hydroflavones reported that chrysin suppressed the level of IL-1β m-RNA in LPS/IFN-γ (lipopolysaccharide/interferon gamma) activated RAW 264.7 mouse macrophages, whereas the addition of OH groups in the C ring (galanin) and B ring (quercetin and kaempferol) decreased this effect [82]. We showed that apigenin reduces TNFα and IL-1β expression in macrophages, even when added after inflammatory stimuli, suggesting its therapeutic potential [52]. Apigenin also reduces nitric oxide synthase (NOS) and cyclooxygenase (COX) expression in macrophages [83,84]. Furthermore, using transgenic NFκB-luciferase reporter mice, we showed that apigenin decreases NFκB activity in vivo [85]. Apigenin was also found to induce apoptosis in mouse macrophage ANA-1 cells through the increased ROS accumulation and high caspase-3 activity followed by activation of the mitogen-activated protein kinase (MAPK) pathway [86]. However, the concentrations used in cellular and animal models are frequently unreachable in vivo, potentially disguising the main mechanisms and clinical potential (Table 2). Apigenin and quercetin inhibited the expression of TNFα in peripheral blood mononuclear cells (PMBC) by halting the NFκB pathway [87,88]. Citrus flavonoid, hesperidin, reduced the ROS generation in human neutrophils and induced caspase-3-dependent apoptosis [89,90]. Flavonoids like epigallocatechin gallate (EGCG), found in green tea, can suppress human monocytic derived dendritic cells through apoptosis and the suppression of cell surface molecules and antigen presentation, suggesting its potential for immuno-therapies [91,92]. Luteolin inhibited lipopolysaccharide induced COX-2 expression and xanthine oxidase generated superoxide in the RAW 246.7 macrophages [93]. In a study pertaining to the effect of flavonoids on macrophage activation, it was shown that flavonols with 3’ and 4’ hydroxylations in the B ring, as in quercetin and kaempferol, exerted the highest degree of TNFα inhibition. Whereas, flavones like apigenin, luteolin and diosmetin were more effective in suppressing the NO and IL-1β production when compared to the flavonols [94,95]. Overall, these findings suggest that flavonoids can affect tumors and its metastases by affecting other components of the TME ecosystem.

### 4.2. Flavonoid-Protein Interactions as Regulators of Breast Cancer

Recent studies provide evidences that flavonoids can also exert their functions by targeting proteins directly. Their structural resemblance to certain estrogens is responsible for the ability of isoflavones (e.g., genistein) and flavanone (e.g., naringenin) to bind to the nuclear estrogen receptors ERα and ERβ [120]. Their increased affinity for ER relies on the 7-OH of A ring and 4′-OH of B ring. However, glycosylated flavonoids like naringenin-7-*O*-glucoside have weaker affinity for ER. Flavonoids have also been found to act as competitive inhibitors of drug transporters, including the breast cancer receptor protein (BCRP) and the ABC transporters P-glycoprotein (P-gp) and multidrug resistance-associated proteins (MRP1 and MRP2) [121,122,123]. Structural based studies of flavonoids on MCF-7 cell lines resistant to the antineoplastic antibiotic mitoxantrone found that apigenin and chrysin increased the accumulation of mitoxantrone in BRCP overexpressing the MCF-7 cells [96]. Similar studies have confirmed the high inhibitory activity of *O*- and *C*-methoxylated flavonoids, such as retusin and ayanin, in MCF-7 BRCP overexpressing cells, underscoring the potential role of the methyl groups in targeting proteins [121,124]. The ability of flavonoids to induce the cell cycle arrest can be attributed to the interaction of its different functional groups with cyclin dependent kinases (CDKs). Molecular dynamic simulations to understand the binding behavior of flavonoids that inhibit CDK6/cyclin D complex showed that hydroxyl groups at 3′ and 4′ positions of B ring (e.g., luteolin) are favorable for the hydrogen bond formation with CDK6/cyclin D, in contrast to the 3-OH on the C ring found in galangin and the 5-OH found in the A ring of chrysin [125]. Apigenin induced G2/M cell cycle arrest in MCF-7 and MDA-MB-468 cell lines by modulating CDK1/cyclin B1 complex accompanied by ERK/MAPK inhibition [126]. Biochemical and molecular docking studies suggested that some methoxyflavones from *Tanacetum gracile* induce cell cycle arrest in MCF-7 and T47D through the direct binding of tubulin [97,127].

The novel approach PD-Seq (phage display coupled with second generation sequencing), that combines the phage display with next generation sequencing, identified 160 direct targets of apigenin from more than 15,000 proteins represented in a human breast cancer library [98]. Notably, in this study our group identified that the top candidate directly interacting with high affinity to apigenin is the heterogeneous ribonuclear protein A2 (hnRNPA2). HnRNPA2 is a RNA binding protein responsible for regulating RNA stability, splicing, microRNA maturation and the recently reported gene expression [98]. HnRNPA2 can participate in enhancing the tumor potential of cells by directly regulating genes involved in resistance to apoptosis, inflammation, and metastasis [128,129,130,131]. We demonstrated that apigenin inhibits hnRNPA2 dimerization affecting its splicing activity. Notably, MDA-MB-231 cells treated with apigenin show splicing isoforms found in non-tumor cells, suggesting the potential of apigenin to reeducate the aberrant tumor-proteome. It would be interesting to see whether the ability of apigenin to interact with hnRNPA2 increases chemosensitization by overcoming the increased anti-apoptotic protection of tumor cells. Intriguing, hnRNPA2 was found to regulate the splicing of the pyruvate kinase (PKM2/PKM1), suggesting the ability of apigenin to regulate central metabolic hubs responsible for the Warburg’s effect [132,133,134,135,136]. Apigenin also directly binds to MUC1 (Mucin 1), a key oncogene participating in tumor growth and metastasis [103,104]. MUC1 is also known to regulate metabolic reprogramming in the MDA-MB-231 cells by altering glutamine dependency of the cells which can be ascribed to the changes in glutamine metabolism [137]. Recent studies also showed that luteolin binds to the transcription factor HNF4α (Hepatocyte Nuclear Factor 4 alpha), repressing fat metabolism [138]. Therefore, understanding these novel mechanisms of action of flavonoids should provide important insights into their prevention and therapeutic potential in breast cancer.

## 5. Dietary Flavonoids and Breast Cancer

Epidemiological studies and systematic analysis suggest that diets rich in flavonoids are inversely associated with BC risk [7,139,140,141,142,143]. The ability of flavonoids to modulate not only cancer cells but also other components of the TME has attracted great interest in the field, providing unique opportunities for the prevention and treatment of BC [5,144]. The health beneficial effects of flavonoids in cancer have been mainly focusing on the cancer cells, seeking to understand the regulation of tumor related genes, signaling pathways, metastasis, and resistance to apoptosis [145,146,147,148,149,150]. However, the breakthrough that flavonoids can modulate the TME provides unforeseen opportunities to halt tumor development, progression and metastasis [151,152,153,154]. Adipocytes, major constituents of BC TME, and its cross talk with cancer cells have strong implications in BC progression, invasion [155,156], induction of aberrant gene expression profiles [157] and therapeutic resistance [158]. Importantly, flavonoids can inhibit adipogenesis, reduce obesity and obesity-related cancer [117,118,159,160]. In addition, adipocytes and macrophages contribute to the tuning of TME making them potential participants of obesity linked cancer and also promising targets for flavonoids [161]. The modulation of T cells by apigenin [100] and dendritic cells (DC) by quercetin [101] can be crucial for overcoming the immunosuppressive condition and inflammation [162,163]. The ability of flavonoids like quercetin and luteolin to target macrophages either by reducing their recruitment into the TME or by reprogramming TAMs from a M2 (tumor promoting) to M1 (tumor suppressive) phenotype has great implications in anti-cancer therapy [112,113,164,165]. Thus, this ability of flavonoids to modulate not only the cancer cells but also other TME accomplices gives them unique opportunities for BC prevention and therapeutic.

### 5.1. Potential Role of Flavonoids in Breast Cancer Therapeutics

Flavonoids have become widely studied in cellular models as a therapeutic agent owing to its low systemic toxicity and broad range of ant-carcinogenic activities. Cancer cells are adaptively resistant to chemotherapeutic drugs, which eventually engender multidrug resistant cells leading to the aggressive tumor growth and metastasis. Sensitization to chemotherapeutic drugs using naturally occurring flavonoids has become an approach of interest aiming to enhance the efficacy of cytotoxic effects, delay the incidence of acquired chemoresistance and halt proliferative pathways [166,167]. Inhibition of transporters like Pgp or MRP by flavonoids like quercetin and luteolin has resulted in an increased bioavailability of cytotoxic drugs, with a potential impact in the quality of life of BC patients [5,168]. Several studies using cellular models have provided strong evidence that flavonoids increase the efficacy of current therapies. Apigenin reduced the expression of MDR1, MRPs and P-gp in MCF-7-doxorubicin resistant cell lines by attenuating STAT3 signaling pathway [102]. Rutin increased the cytotoxicity of cyclophosphamide and methotrexate on MDA-MB-231, MCF-7 and primary human mammary fibroblasts (HMF) in a time dependent manner, by suppressing the expression of P-gp and BCRP, arresting the cells at G2/M and G0/G1 phases and promoting cell apoptosis [103]. Isoflavones also effectively inhibit BCRP [122]. Epigallocatechin gallate with tamoxifen has shown to synergistically enhance the cytotoxic effect against MDA-MB-231 cell lines **[104]**. Resveratrol chemosensitization to doxorubicin functioned by hindering NFκB andby impeding the expression of Hsp27, an inhibitor of caspase-3, in MCF-7 cell lines [169,170,171]. The resveratrol-doxorubicin combination also showed a 2.5 fold of dose advantage in inhibiting growth, NFκB activity and inducing apoptosis in MDA-MB-231 and MCF-7 cell lines [105]. Apigenin enhanced cisplatin cytotoxicity through a p53 mechanism in MCF-7 cells [106]. Flavopiridol, a semisynthetic flavone and trastuzumab synergistically inhibited the P13k/Akt pathway and induced cell cycle arrest in SK-BR3 (HER2+) and BT-474 (ER+) cell lines [107]. It was also found to enhance the cytotoxicity induced by sorafenib, a Raf inhibitor, in MDA-MB-231, MDA-MB-468 and SK-BR3 cell lines. Interestingly, this synergy also reduced the primary tumor growth rates and metastatic tumor load in the lungs of MDA-MB-231 mammary fat pad engraftment mouse models, compared to treatment with either drug alone [108]. However, the combinational treatment of flavopiridol with docetaxel and trastuzumab at clinical levels did not report any significant effect. While quercetin or doxorubicin alone failed to cure tumor-bearing mice, a combination regime induced significant depletion of 4T1 BC and led to a long-term, tumor-free survival in mice bearing established breast tumor along with persistent T-cell tumor-specific responses [114]. These findings suggest that the anti-carcinogenic activities of flavonoids might be more effective in combination with other chemotherapeutic agents than when used alone.

Mechanistically some flavonoids have been shown to increase the expression of receptors of therapeutic drugs. Flavones like chrysin [172], luteolin [173] and apigenin [174] have increased the expression of TRAIL receptor DR5 and thereby contributing to TRAIL induced and chemotherapeutic induced cytotoxicity in multiple cancer cell lines by inhibiting the NFκB pathway and activation of caspase-3, -8, -9 and -10. Thus, flavonoid and TRAIL combination treatments can be a promising therapy against malignant breast tumors. As potent apoptosis inducers, flavonoids are known to possess cytotoxicity against cancer cells. Apigenin can induce apoptosis through caspase activation, PARP cleavage and pro- and anti-apoptotic markers Bax/Bcl-2 ratio [109]. In BT-474 and MDA-MB-231 xenograft mice models, apigenin attenuated growth through the induction of apoptosis and inhibition of proteasome activity and VEGF [115,116]. Interestingly, wogonin, chrysin and apigenin were shown to induce TRAIL mediated apoptosis in MDA-MB-231 by downregulating cellular FLICE-like inhibitory protein (c-FLIP) expression and enhancing the expression of TRAIL DR5 [110]. We have earlier reported that apigenin, through its direct interaction with hnRNPA2, can reduce the levels of splice isoform of c-FLIP (c-FLIP_S_) and caspase-9 (caspase-9b) in MDA-MB-231, two key RNA isoforms that when translated play a role in inhibiting apoptosis in cancer cells [98]. These findings together uphold the therapeutic effect of flavones such as apigenin in breast cancer cells, emphasizing the need for more studies in xenograft and preclinical models. Current research is also investigating the tuning of TME cells with the aid of flavonoids in order to enhance the effect of the chemotherapeutic drugs. Cellular studies show that genistein enhances the natural killer cell mediated cytotoxicity in MCF-7 cell lines [175,176]. Hence, there is an increasing need to identify chemosensitizers targeting preferentially to the tumor site that can provide low systemic toxicity and enhanced therapeutic drug efficacy. This clearly suggests the potential benefit of flavonoids as a chemosensitizer and an immunomodulator and hence emphasizes the need for combinational therapy as an approach to treat BC.

### 5.2. Potential Role of Flavonoids in Breast Cancer Prevention

Several epidemiological studies have shown that flavonoid rich diets are associated with the reduction of cancer risk in humans [177]. Mediterranean and Asian diets, which are highly rich in flavonoids, are often linked to a reduced risk of BC [178,179,180]. Increased consumption of isoflavones decreased the risk of estrogen-related cancers [181,182]. In a meta-analysis of 12 prospective cohort or case-control studies, the risk of BC was shown to be significantly decreased in women with a high intake of flavonols and flavones among post-menopausal women [141]. The Shanghai Women’s Health Study showed higher levels of urinary epicatechins relating to a lesser incidence of breast cancer [183]. Similarly, several case control studies have reported that an increased intake of flavones can reduce the BC risk in women [139,140,143].

Obesity is reaching epidemic levels across the world [184]. Approximately 35% of the adult US population is overweight [185]. Obesity is often associated with a higher risk of developing BC and worse disease outcomes for women of all ages, irrespective of menopausal status [186]. In the Breast Cancer Prevention P-1 trial and World Health Initiative trial I, two among the many clinical trials conducted on obesity-BC relation, found that obesity was strongly associated with higher premenopausal [187] and postmenopausal BC risk [188], respectively. The Million Women Study, which included 45,037 breast cancer women between the ages of 50–64 years, identified a 30% higher risk of developing postmenopausal breast cancer with obesity in UK women [189]. However, studies have shown that the risk for TNBC is higher among premenopausal women. The Cancer and Steroid Hormone, a population-based case-control study involving 3432 BC patients, reported a strong positive association between body mass index (BMI) and premenopausal TNBC risk [190]. Similarly, two meta-analyses of 1358 [191] and 620 [192] TNBC patients showed an 80% and 43% higher risk of developing TNBC in obese premenopausal women, respectively. Adipose tissues secrete cytokines and mediators which participate in creating an environment that favors cancer invasion and metastasis and are highly amplified in obese individuals [193]. Increased inflammatory condition, a characteristic of obesity, has also been largely associated to increased incidences and poor clinical outcomes [194,195,196]. Obese individuals display dysregulated genes expression, harboring TME inflammatory conditions that increase macrophage infiltration and adipokine imbalance in breast tissues [196,197,198]. Interestingly, a recent retrospective study showed a relation between obesity and the development of metastasis in BC patients and indicated that non-obese patients showed a better response to first-line metastatic chemotherapy treatment when compared to obese patients [199]. Investigations on flavonoids as potent agents against obesity and obesity-linked BC are gaining great interest. A study involving 9551 adults who participated in the National Health and Nutrition Examination Survey 2005–2008 reported that flavonoid consumption was inversely related to obesity in multivariate models of both men and women with significant reduction in BMI and C-reactive protein, a marker for inflammation, in US adults. Flavonoids from wheat (*Triticum aestivum*) sprout showed an inhibitory effect on adipogenesis through the downregulation of genes associated with lipid metabolism and adipogenic transcription factors [200]. Adipocytes are known to be potent modulators and recruiters of TAMs, thereby actively contributing to tumor growth. Luteolin strongly inhibited the interaction between 3T3-L1 adipocytes and RAW246 macrophages, thereby suppressing the production of pro-inflammatory mediators like TNF-α [111]. In animal models, flavonoid-enriched extract from *Hippophaerhamnoides L.* seed induced anti-obesity activities in high fat diet (HFD) C57BL/6 mice and recruited macrophage infiltration into the adipose tissues [118]. Naringenin not only reduced adipose mass and inflammation but had a moderate effect on the inhibition of tumor growth in C57BL/6 mice injected with E0771 mammary carcinoma cell line [127]. Resveratrol was reported to inhibit obesity-associated inflammation and claudin-low BC growth in diet induced obese C57BL/6 mice by inhibiting adipocyte hypertrophy and associated adipose tissue dysregulation [119]. Interestingly, two recent independent studies provided preclinical evidence using MMTV-PyMT murine models of BC that mammary adipose tissue inflammation induced by diet enhanced the recruitment of macrophages, increased tumor vascular density [201] and hormone production in pre- and postmenopausal hormone receptor positive BRCA [202], suggesting a role for obesity in creating a microenvironment favorable for the progression of breast cancer. However, there is a scarcity of data on the effect of flavonoids on HFD induced mammary tumorigenesis in preclinical PyMT mouse models, which can be highly corroborative for clinical studies targeting cancer prevention.

## 6. Clinical Applications of Flavonoids: Challenges and Opportunities

The increasing need to find more effective preventive and therapeutic approaches to treat and control BC has led to deepened studies on the role of flavonoids. This is particularly important in TNBC where poor survival rates and lack of effective therapies have prompted alternative preventive and therapeutic strategies. The scarce case control and cohort studies conducted so far indicate that a flavonoid rich diet can efficiently modify BC (Table 3). In a study involving 820 women from Greece, the regular intake of vegetables rich in flavones showed the correlation to a reduced risk of BC [143]. Patient and population-based studies on the potential relation of a flavonoid rich diet and risk of BC in women from Italy and Long Island showed a significantly inversed association, especially in postmenopausal women [139,140]. Interestingly, the high consumption of tea reduced the risk of ER negative BC in women who participated in the Cancer Prevention Study-II Nutrition Cohort owing to its rich flavan-3-ol content [203]. These findings have helped to establish a promising platform for treatment of BC with a high flavonoid diet. Soy protein food supplements rich in isoflavones have been of increased interest in treating women at high risk for or with BC. A meta-analysis of 35 studies including pre- and post-menopausal women reported that soy isoflavone intake can significantly lower BC risk in Asian women [180]. Soy intake after diagnosis of BC (including ER+, PR+, both and TNBC) was significantly associated with a reduced risk of recurrence in a large study of ~9500 women from US and China [204]. In a similar small-scale study with 358 incident BC patients from Korea, an estimated intake of 15 mg/day isoflavones and 77 g/day soy showed an inverse association among postmenopausal women albeit no difference with respect to the ER+/PR+ status [205]. Findings in the previous section have confirmed that obesity increases the risk of BC development and relapse. A recent pilot study to test the feasibility of a 12-week soy-based meal replacement weight loss intervention among ER/PR negative BC survivors showed that an isoflavone rich diet reduced the body weight, total cholesterol and may impact the risk of BC recurrence [206]. However, a six-month intervention of soy isoflavones supplements in high risk adult western women showed no reduction in breast epithelial proliferation and, instead, had adverse effects in premenopausal women [207]. This could suggest that the use of dietary rich flavonoids seem warranted for BC control. Table 4 lists some of the completed clinical trials conducted on BC patients with flavonoids. Although several clinical trials involving flavonoids like genistein, soy isoflavones and flavopiridol have been successful in establishing favorable dose ranges without any adverse effects in patients, there is no promising data supporting the inhibitory effect of diet rich in these compounds on BC [208,209,210]. The absence of any profound effect from the combinational treatment with flavonoids and chemotherapeutic drugs points out that it demands more than just the mere establishment of effective dose range [208,211,212].

Despite the numerous studies towards the development of new prevention and therapeutic strategies and preclinical trials, there is an increasing need for more warranted long term and population-based studies. Studies involving large cohorts covering a wide range of criteria must be considered for clinical trials. The benefits of flavonoids for chemoprevention in clinical trials have come across only restricted success, mainly owing to its varying bioavailability and ineffective systemic delivery, and thereby hindering in reaching effective concentrations. As reported in Table 2, flavonoids mostly exert cytotoxic effects only at moderately high doses, within a micromolecular concentration range [229]. Numerous in vitro studies demonstrating a constructive effect use doses that are several magnitudes higher than what the cells can essentially tolerate in vivo. This has posed a critical concern in establishing the effect of flavonoids in clinical trials. However, this can be overcome by using physiologically and clinically relevant concentrations and regimens of flavonoids in the in vitro and preclinical studies. Future studies addressing the bioavailability and efficacy of in vivo concentrations are of paramount significance to advance our understanding of how flavonoids exert their health beneficial effects.

The level of dietary flavonoids in the plasma differs in accordance with structural disparities, concentration and source. Nevertheless, attaining levels in plasma that would be sufficient to activate the anti-cancer effects through oral administration is indeed challenging. It is highly likely that these phytochemicals accumulate in the intestine and colon during systemic metabolism at higher levels than in plasma and thereby affecting its bioavailability. Apart from the structural characteristics of flavonoids, gut microbiota also plays a significant role in the metabolism of flavonoids [230]. The gut microbiota not only modifies the structure of the parent aglycones, conjugates and glycosides but also catabolizes chemical changes such as dihydroxylation, decarboxylation and hydrogenation of the compounds to generate smaller phenolic acids, which are highly bioavailable [231]. Some of these metabolites have higher retention levels in plasma compared to their parent compounds. For example, the plasma level of cyanidin glucosides is 0.02% while that of its microbiota dependent metabolite 3-4-dihydroxybenzoic acid is 44% [232]. Much attention needs to be paid to the activities and availability of these metabolites. The absorption level of such metabolites degraded in the large intestine seems to be higher than that of the parent compound thereby being more effective [233]. Hence, bioavailability, microbiome and its governing factors must be taken into consideration in order to provide vital insights into food and clinical conclusions. Also, the anti-carcinogenic activity of the flavonoids would be contingent not only upon the flavonoid itself, but also on its metabolites, which makes the identification and quantification of these conjugates crucial in understanding the health benefits of dietary flavonoids in cancer patients. In addition, in accordance to the findings mentioned in the previous sections, the effect of flavonoid rich meal regimens on immune regulation must be considered for clinical trials, which can eventually be eye openers to new treatments.

Furthermore, flavonoid metabolism can also be influenced by substantial inter-individual variations between the participants in the study, thereby demanding nutrigenomic approaches to ameliorate therapy [234]. It could be possible that the genomic variation in individuals can contribute to different retention levels and metabolism of flavonoids. The discrepancies pertaining to the effect of flavonoids observed in clinical trials could be explained with the help of individual variability with regards to intestine microbiota composition and gene polymorphisms [235]. Consequently, understanding nutrigenomics can help in establishing the individual nutritional requirements based on genetic makeup of the person as well as the relationship between diet and cancer. Dietary supplements as an alternative source of flavonoids have become prevalent in the last decade. However, there is insufficient data on the assessment if anticancer characteristics of flavonoid rich diet can be replaced with purified compounds such as flavonoid supplements. The food sources of flavonoids may include mixtures of secondary metabolites from plants which may not be true for the single compound dietary supplements [236]. Moreover, drug to nutrient and drug-to-drug interactions along with toxicity issues need to be considered for supplements. Therefore, there is a partial recognition of essentials and requirements for intervention studies which is a barrier to achieving the goal of evaluating the health benefits in clinical trials and developing dietary recommendations for flavonoid intake to prevent and treat cancer.

## 7. Conclusions

The value of flavonoids in health is well accepted, yet their role in BC prevention and treatment remains unclear. Conflicting reports and the limited number of clinical studies may have a great impact in the future utilization of this untapped natural compound in the clinical set up. Increasing our understanding of flavonoid structure, metabolism and molecular activities is paramount for the implementations of more effective clinical studies. Much has been learnt using cellular systems and pure flavonoids. As we move into the future, the field needs more investigations; broadened use of preclinical models, test effects of nutritional achievable doses and delivery systems compatible for human studies. The implementation of these strategies would accelerate the transition of using flavonoids in the clinic for the prevention and treatment of breast cancer.

## Figures and Tables

**Table 1:** Breast cancer (BC) molecular subtypes.

Subtype	Biomarker Status	Prognosis
Luminal A	ER+ and/or PR+, HER2−, Ki67−	Good
Luminal B	ER+ and/or PR+, HER2−, Ki67+ ER+ and/or PR+, HER2+, Ki67+	Medium
HER2 enriched	ER−, PR−, HER2+	Poor
Basal-like	ER−, PR−, HER2−, basal markers (keratin 5, 6, 14, 17, EGFR etc.)	Poor
Normal-like	ER+ and/or PR+, HER2−, Ki67−	Medium

ER: Estrogen receptor; HER2: Human epidermal growth factor receptor; PR: Progesterone receptor; Ki67: Proliferation marker protein; EGFR: Epidermal growth factor receptor.

**Table 2:** Flavonoid doses in different experimental models.

Study Type	Experimental Model	Treatments	Dose	Results	Reference
Cellular Studies	T47D and MCF-7	Wine polyphenols	1 pM–100 nM	Inhibited growth and antagonize H_2_O_2_	[56]
	HCC70, BT-474 and T47D	Butein	0.001–100 μg/mL	Induced apoptosis through ROS reduction	[58]
	MDA-MB-468	Naringenin	2.5–50 μM	Oxidative stress induced apoptosis	[59]
	MDA-MB-231 and MDA-MB-468	Myricetin	20–100 μM	Oxidative stress induced apoptosis	[60]
	MDA-MB-453	5,7-dihydroxy, 8-nitrochrysin	2–8 μM	Induced apoptosis by generation of ROS	[63]
	MDA-MB-231	Silibinin	30 μM	Induced apoptosis	[64]
	Human monocytes and RAW 264.7	Apigenin	0.1–25 μM	Reduced NFκB phosphorylation, TNF-α and IL-1β expression	[52]
	RAW 264.7	Chrysin	30 μM	Suppression IL-1β expression	[82]
	RAW 264.7	Apigenin	10–25 μM	Reduced NOS and COX expression	[83]
	ANA-1	Apigenin	12.5–200 μM	Induced apoptosis	[84]
	Human PBMC	Quercetin	1–50 μM	Inhibited TNF-α expression	[87]
	Human Neutrophils	Hesperidin	1–100 μM	Reduced ROS generation and induced apoptosis	[90]
	Human Dendritic cells	EGCG	10–100 μM	Induced apoptosis	[92]
	RAW 264.7	Luteolin	25–100 μM	Inhibited COX-2 and xanthine oxidase expression	[93]
	Bone marrow derived mouse macrophages	Quercetin and Kaempferol	25–50 μM	Inhibited TNF-α expression	[95]
	MCF-7	Apigenin and Chrysin	10–50 μM	BCRP inhibitors	[96]
	MCF-7 and T47D	Methoxyflavones from *Tanacetum gracile*	1.5–5 μM	Induced cell cycle arrest through tubulin binding	[97]
	MDA-MB-231	Apigenin	25 μM	Inhibited hnRNPA2 dimerization affecting its splicing activity	[98]
	MCF-7	Apigenin	25–100 μM	Suppressed MUC-1 expression and induced apoptosis	[99]
Cellular Study	CD4 T cells	Apigenin	12.5–75 μM	Potentiated activation induced cell death by suppressing NFκB regulated anti-apoptotic pathways	[100]
	Dendritic cells	Quercetin	50 μM	Attenuated LPS induced DC activation	[101]
	MCF-7	Apigenin	20–80 μM	Reduced cell growth and expression of MDR1 and P-gp in MCF-7-doxorubicin resistant cells	[102]
	MCF-7, MDA-MB-231 and HMF	Rutin	20 μM	Increased the cytotoxicity of cyclophosphamide and methotrexate	[103]
	MDA-MB-231	EGCG	10–25 μM	Synergistic enhancement of cytotoxicity with tamoxifen	[104]
	MCF-7	Resveratrol	50–250 μM	Increased sensitivity to doxorubicin	[79]
	MDA-MB-231 and MCF-7	Resveratrol	80–180 μM	Synergistic inhibition of growth with doxorubicin	[105]
	MCF-7	Apigenin	30 μM	Enhanced cisplatin cytotoxic activity	[106]
	BT-474 and SK-BR3	Flavopiridol	50–100 nM	Synergistic inhibition of cell proliferation with trastuzumab	[107]
	MDA-MB-231, MDA-MB-468 and SK-BR3	Flavopiridol	0.2 μM	Enhanced sorafenib induced cytotoxicity	[108]
	BT47D and MDA-MB-231	Apigenin	10–80 μM	Induced apoptosis and autophagy	[109]
	MDA-MB-231	Wogonin	50–100 μM	Sensitized TRAIL-induced apoptosis	[110]
	RAW 264.7 macrophages and 3T3-L1 adipocytes co-culture	Luteolin	1–20 μM	Suppressed the adipocyte-dependent activation ofmacrophage	[111]
3D Study	MDA-MB-231	Apigenin and Luteolin	20 μM	Attenuate growth and intravasation through endothelial barrier	[69]
Animal Study	BALB/C-Tg (NFκB-RE-luc)-Xen mice	Apigenin	50 mg/kg body weight	Reduced NFκB activity in lungs in vivo	[85]
	CD-1 immunodeficient mice bearing MDA-MB-231 tumor	Oncamex	25 mg/kg body weight	Inhibited tumor growth	[67]
	Athymic nu/nu nude mice bearing MDA-MB-231 tumors	Radix Glycyrrhiza extracts	20–100 mg/kg body weight	Attenuated tumor growth through iNOS inhibition	[68]
	C57BL/6 mice	Luteolin	HFD with 0.01% luteolin	Inhibited inflammatory macrophage polarization in adipose tissue	[112]
	C57BL/6 mice	Quercetin	HFD with 0.1% luteolin	Attenuated macrophage recruitment and modulated M1/M2 macrophage ratio	[113]
	BALB/c mice bearing 4T1 tumors	Quercetin	5mg/kg body weight	Synergistic inhibition of tumor growth with doxorubicin	[114]
	Athymic nu/nu nude mice bearing BT47D tumors	Apigenin	50 mg/kg body weight	Inhibited the progression progestin dependent BT-474 xenograft tumors in nude mice through apoptosis	[115]
	Athymic nu/nu nude mice bearing MDA-MB-231 tumors	Apigenin	25–50 mg/kg body weight	Inhibited tumor proliferation and proteasome activity	[116]
	Ovariectomized C57BL/6 mice injected with E0771 cells	Naringenin	HFD with 1–3% naringenin	Reduced adipose tissue mass and ameliorated adipose tissue inflammation	[117]
	C57BL/6 mice	*Hippophae rhamnoides* L. seeds extracts	100–300 mg/kg body weight	Significant anti-obesity and anti-inflammatory effect	[118]
	Ovariectomized female C57BL/6 mice	Resveratrol	300–600 mg/kg body weight	Inhibited obesity-associated increases in claudin-low mammary tumor growth and macrophage infiltration	[119]

BRCP: Breast cancer receptor protein; hnRNPA2: heterogeneous nuclear ribonuclear protein A2; MUC-1: Mucin-1; DC: Dendritic cells; MDR1: Multidrug resistance 1; P-gp: P-glycoprotein; TRAIL: TNF-related apoptosis-inducing ligand; HFD: High fat diet.

**Table 3:** Epidemiological prospective cohort and case control studies on dietary intake of flavonoids and breast cancer risk.

Subclass of Flavonoid	Study Type	Region	Cases/Controls	Association with BC Risk	Reference
Flavones	Hospital based	Greece	820/1548	Inverse	[143]
Flavonols, Flavones	Hospital based	Italy	2569/2588	Inverse	[139]
Flavones	Population-based	New York	1434/1440	Inverse	[140]
Flavonoids	Cohort	America	1351/38,408	No effect	[213]
Isoflavones	Cohort	Singapore and China	629/35,303	Inverse	[214]
Isoflavones	Cohort	Japan	134/15,264	Inverse	[215]
Flavonols, Flavones	Hospital based	Mexico	141/141	Inverse	[216]
Isoflavones	Population-based	Canada	3024/3420	Inverse	[217]
Isoflavones	Hospital-based	Korea	358/360	Inverse	[205]
Flavonoids	Cohort	Europe	11,576/334,850	No effect	[218]
Flavone, Flavan-3-ol	Cohort	America	56,630	Inverse	[203]
Isoflavones	Population-based	England	240/477	Inverse	[219]
Isoflavones	Hospital-based	Japan, Brazil	390/390	Inverse	[220]
Isoflavones	Hospital-based	China	438/438	Inverse	[221]
Flavonoids	Cohort	Dutch	199/3209	Inverse	[222]
Isoflavones	Cohort	Multiethnic	4769/84,450	No effect	[223]

**Table 4:** Clinical trials on dietary intake of flavonoids and breast cancer risk.

Flavonoid Subclass/Name	Study Type	Detail	Enrollment	Comments	ClinicalTrials.gov Identifier
Genistein (with Gemcitabine)	Phase II	Stage IV BC	17	No effect	NCT00244933 [211]
Genistein	Phase II	NA	126	No effect	NCT00290758 [207]
Genistein	Phase I	NA	30	Dose rangestudy	NCT00099008 [210]
Catechin	Phase I	HER2-, stage I-III BC	40	Dose range study	NCT00516243 [224]
Catechin	Phase II	NA	1075	No significant effect	NCT00917735 [225]
P276-00, flavone derived	Phase I	TNBC	11	NA	NCT01333137 [226]
Soy Isoflavone	Phase II	Post-menopausal	NA	NA	NCT00200824 [227]
Soy Isoflavone	Phase III	Pre-menopausal	100	No adverse effect	NCT00513916 [209]
Soy Isoflavone	NA	BRCA1 & 2 stage I-III BC	110	No effect	NCT01219075 [228]
Flavopiridol (with Trastuzumab)	Phase I	HER2+, stage IV BC	50	Dose range study	NCT00039455 [208]

NA: Not available.
